# A Latent Class Analysis of Perceived Barriers to Help-seeking Among People with Alcohol Use Problems Presenting for Telephone-delivered Treatment

**DOI:** 10.1093/alcalc/agac063

**Published:** 2022-11-30

**Authors:** Jasmin Grigg, Victoria Manning, Ali Cheetham, George Youssef, Kate Hall, Amanda L Baker, Petra K Staiger, Isabelle Volpe, Peta Stragalinos, Dan I Lubman

**Affiliations:** Turning Point, Eastern Health, Church St Richmond, 3121, Australia; Monash Addiction Research Centre, Eastern Health Clinical School, Moorooduc Hwy Melbourne, 3199, Australia; Turning Point, Eastern Health, Church St Richmond, 3121, Australia; Monash Addiction Research Centre, Eastern Health Clinical School, Moorooduc Hwy Melbourne, 3199, Australia; Turning Point, Eastern Health, Church St Richmond, 3121, Australia; Monash Addiction Research Centre, Eastern Health Clinical School, Moorooduc Hwy Melbourne, 3199, Australia; School of Psychology, Deakin University, Pigdons Rd Geelong, 3216, Australia; Centre of Drug, Addictive and Anti-social Behaviour Research (CEDAAR), Deakin University, Burwood Hwy Melbourne, 3125, Australia; Centre for Adolescent Health, Murdoch Children’s Research Institute, Flemington Rd Melbourne, 3052, Australia; School of Psychology, Deakin University, Pigdons Rd Geelong, 3216, Australia; Centre of Drug, Addictive and Anti-social Behaviour Research (CEDAAR), Deakin University, Burwood Hwy Melbourne, 3125, Australia; School of Medicine and Public Health, University of Newcastle, University Drv Callaghan, 2308, Australia; School of Psychology, Deakin University, Pigdons Rd Geelong, 3216, Australia; Centre of Drug, Addictive and Anti-social Behaviour Research (CEDAAR), Deakin University, Burwood Hwy Melbourne, 3125, Australia; Turning Point, Eastern Health, Church St Richmond, 3121, Australia; Monash Addiction Research Centre, Eastern Health Clinical School, Moorooduc Hwy Melbourne, 3199, Australia; Turning Point, Eastern Health, Church St Richmond, 3121, Australia; Monash Addiction Research Centre, Eastern Health Clinical School, Moorooduc Hwy Melbourne, 3199, Australia; Turning Point, Eastern Health, Church St Richmond, 3121, Australia; Monash Addiction Research Centre, Eastern Health Clinical School, Moorooduc Hwy Melbourne, 3199, Australia

## Abstract

**Aims:**

Despite the magnitude of alcohol use problems globally, treatment uptake remains low. This study sought to determine the proportion of people presenting to telephone-delivered alcohol treatment who are first-time help-seekers, and explored perceived barriers to help-seeking to understand the barriers this format of treatment may help to address.

**Methods:**

Secondary analysis of baseline data from a randomized controlled trial of a telephone-delivered intervention for alcohol use problems. Latent class analysis (LCA) identified participant profiles according to self-reported barriers to alcohol treatment.

**Results:**

Participants’ (344) mean age was 39.86 years (SD = 11.36, 18–73 years); 51.45% were male. Despite high alcohol problem severity (Alcohol Use Disorder Identification Test: mean = 21.54, SD = 6.30; 63.37% probable dependence), multiple barriers to accessing treatment were endorsed (mean = 5.64, SD = 2.41), and fewer than one-third (29.36%) had previously accessed treatment. LCA revealed a two-class model: a ‘low problem recognition’ class (43.32%) endorsed readiness-for-change and attitudinal barriers; a ‘complex barriers’ class (56.68%) endorsed stigma, structural, attitudinal and readiness-to-change barriers, with complex barrier class membership predicted by female sex (adjusted OR = 0.45, 95% CI 0.28, 0.72) and higher psychological distress (adjusted OR = 1.13, 95% CI 1.08, 1.18).

**Conclusion:**

The majority of people accessing this telephone-delivered intervention were new to treatment, yet had high alcohol problem severity. Two distinct profiles emerged, for which telephone interventions may overcome barriers to care and tailored approaches should be explored (e.g. increasing problem awareness, reducing psychological distress). Public health strategies to address stigma, and raise awareness about the low levels of drinking that constitute problem alcohol use, are needed to increase help-seeking.

## Teaser

**Short Summary:** Most people accessing telephone-delivered treatment for alcohol use problems were new to treatment. Latent class analysis identified two profiles regarding barriers to help-seeking, a ‘low problem recognition’ class, and a ‘complex barriers’ class (predicted by female sex and higher psychological distress), for which telephone interventions may help to overcome barriers to care.

## INTRODUCTION

Alcohol consumption is a leading cause of preventable morbidity and mortality globally, resulting in 5.3% (3 million) of all deaths and 5.1% of all disease burden annually (Griswold *et al*., 2018; World Health Organization, 2022), with alcohol-attributable burden of disease increasing over time (Murray *et al*., 2020). Alcohol use disorders are estimated to affect 5.1% of the adult population worldwide (World Health Organization, 2018), yet rates of treatment uptake remain low in sharp contrast to the magnitude of alcohol consumption and attributable harms. In high income countries, fewer than 10% of people with an alcohol use disorder receive treatment, which is among the lowest treatment coverage of any mental health disorder (Rehm *et al*., 2012). When treatment is sought, delays are substantial, with first treatment contact occurring a median of 18 years following the development of alcohol problems (Chapman *et al*., 2015). From a public health perspective, identifying alternative approaches that overcome obstacles to treatment is imperative to encourage earlier help-seeking.

Low rates of help-seeking for alcohol use problems are associated with multiple individual-level and structural barriers (Oleski *et al*., 2010; Probst *et al*., 2015; Schuler *et al*., 2015; May and Nielsen, 2019; Bernard *et al*., 2020). Studies with treatment-naïve cohorts and those accessing in-person services have shown the most prevalent barrier to alcohol treatment to be low problem awareness (i.e. alcohol consumption is not considered to be problematic or warranting treatment) (Oleski *et al*., 2010; Probst *et al*., 2015; Schuler *et al*., 2015). However, a recent systematic review identified *shame and stigma* to be the most prevalent barrier (May and Nielsen, 2019) followed by low perceived treatment need (operationalized to involve low problem awareness, and perceptions that the problem would resolve itself, was not severe enough to require treatment, and that one should be able to handle the problem alone) (May and Nielsen, 2019). The *desire to keep drinking* (i.e. enjoyment of drinking, not wanting to abstain) was also identified as a prominent barrier preventing access to treatment (May and Nielsen, 2019). *Structural barriers* (i.e. financial constraints, transportation, treatment wait times and geographical proximity to treatment services) were less commonly identified (May and Nielsen, 2019). Moreover, since 2020, access to treatment has been severely disrupted by the COVID-19 pandemic, a global public health emergency that has further increased alcohol consumption and related harms among vulnerable groups (Pollard *et al*., 2020; Sallie *et al*., 2020; Ogeil *et al*., 2021), and which has led to the rapid expansion of telehealth use worldwide in response to government policies aimed at containing virus transmission (e.g. social distancing, lockdowns).

Telephone-delivered interventions have considerable potential to overcome many of the structural (e.g. distance to services, in-person treatment wait times) and individual (e.g. readiness for in-person treatment, fear of shame/stigma) barriers to accessing treatment for alcohol problems. Yet, until now they have been underutilized in substance use populations (Lin *et al*., 2019), other than for promoting smoking cessation (Stead *et al*., 2013). An emerging body of literature provides evidence for the benefits of telephone-delivered interventions for alcohol use problems (Heinemans *et al*., 2014; Gates and Albertella, 2016; Grigg *et al*., 2022); telephone-delivered interventions have been shown to be comparable to in-person treatment in reducing alcohol consumption (Gates and Albertella, 2016), enable experiences of therapeutic alliance and rapport building (Bernard *et al*., 2020), and there is growing evidence that they are filling a gap in service provision for health inequity groups (e.g. women, people living in regional and remote areas) (Grigg *et al*., 2022). However, no known studies have formally investigated the barriers to help-seeking experienced by individuals accessing alcohol treatment via this platform, or whether these more accessible services are engaging people earlier in their treatment trajectories.

Therefore, this study sought to determine the proportion of people presenting to telephone-delivered alcohol treatment who are first-time help-seekers, and utilized latent class analysis (LCA) to identify relatively homogenous and unobserved (i.e. latent) classes of individuals based on perceived barriers to help-seeking for alcohol use problems, to increase our understanding of the barriers this format of treatment may help to address.

## METHODS

### Study design

This was a secondary analysis of baseline data from a randomized controlled trial of a standalone telephone-delivered cognitive and behavioural intervention (Ready2Change; R2C) for individuals with alcohol use problems from the general population (Australian New Zealand Clinical Trials Registry, ACTRN12618000828224) (Lubman *et al*., 2019). The trial was approved by the Eastern Health and Monash University Human Research Ethics Committees. Participants provided verbal informed consent to participate. Assessments were conducted by telephone, with data collected and managed using REDCap (Harris *et al*., 2019). Data for the current study were extracted from the trial dataset in September 2020. Reporting of this study followed the STROBE statement for reporting observational studies (Von Elm *et al*., 2007).

### Participant recruitment and eligibility

The parent trial was conducted at Turning Point, a national addiction treatment and research centre based in Melbourne, Australia. Participants were recruited from across Australia between May 2018 and October 2019 via social media advertising, clinician referrals, and advertising in University and hospital newsletters. Recruitment material invited people to participate who were interested in receiving one of two telephone-delivered support programs to help them reduce their alcohol use. Participants aged ≥18 years with problem alcohol use indicated by an Alcohol Use Disorders Identification Test (AUDIT) (Babor *et al*., 2001) score of >6 (females) or >7 (males) were recruited between May 2018 and October 2019. Individuals with severe alcohol dependence requiring urgent medical treatment, low-risk alcohol consumption, a history of psychosis, active suicidality, an acquired brain injury, attending other alcohol treatment, experiencing substantial hearing impairment, and pregnant women were excluded via initial screening assessment. Full details of the trial have been published (Lubman *et al*., 2019). Participants were reimbursed with an AUD$20 voucher for participating in the baseline assessment. The total sample of participants included in the parent trial (pooled across both arms of the trial) were included in the current study.

### Data collection

Participants’ demographic (i.e. age, sex, culturally and linguistically diverse, Aboriginal and/or Torres Strait Islander descent, geographic area, education, employment status) and clinical information (i.e. alcohol problem severity, past-month drinking patterns, age of first/regular alcohol use, previous alcohol treatment, past-month use of other drugs, psychological distress, barriers to seeking alcohol treatment) were collected. Data collection included use of the following measures: (a) the AUDIT measure of alcohol problem severity (Babor *et al*., 2001), (b) the Timeline Follow-back (TLFB) measure of past-month drinking patterns (Sobell and Sobell, 1992), (c) the Kessler Psychological Distress Scale (K10) (Kessler *et al*., 2003), and (d) the United States’ (US) National Epidemiologic Survey on Alcohol and Related Conditions (NESARC) list of 15 binary indicator variables identifying perceived barriers to seeking treatment for alcohol problems, with each barrier pre-defined as belonging to one of five barrier domains: attitudinal, stigma, readiness for change, financial, and structural (Grant *et al*., 2004; Schuler *et al*., 2015). No time-frame during which barriers were experienced was stipulated (i.e. lifetime barriers).

### Bias

Potential sources of bias were minimized in the following ways (Sterne *et al*., 2016): risk of selection bias was minimized by including in the analyses all participants that had been eligible to participate in the trial; the researcher conducting baseline assessments was trained and experienced in the administration of the psychometric measures, reducing the risk of measurement bias; and, measurement bias was further minimized by using validated, structured measures when available.

### Statistical analyses

Estimated resident population (ERP) data for Australia were used to calculate the rate of participation by geographic remoteness area (Australian Bureau of Statistics, 2018). ERP counts for each remoteness classification were averaged over calendar years 2018–2019 to provide an average population estimate by geographic area, collapsed to (a) metropolitan: major cities of Australia; (b) non-metropolitan: inner regional Australia, outer regional Australia, remote Australia, and very remote Australia. Rates of participation by metropolitan/non-metropolitan area were provided as rate per 100,000 population. Summary data were presented as counts and percentages for categorical variables, and means and standard deviations (SD) for numeric variables. Analysis of variance (ANOVA) and Pearson’s chi-squared tests were used to examine associations between trial eligibility/non-eligibility and demographic/clinical variables.

Latent class analysis (LCA) was performed to identify latent classes of individuals with similar response patterns on the NESARC’s set of 15 binary indicator variables addressing specific barriers to alcohol treatment (Grant *et al*., 2004; Schuler *et al*., 2015). To determine the optimal number of latent classes, LCA models of increasing sizes were sequentially estimated, with model fit examined using Akaike information criterion (AIC) (Akaike, 1987), Bayesian information criterion (BIC) and adjusted BIC (Schwarz, 1978), and Lo–Mendell–Rubin likelihood ratio test (LMR LRT) (Lo *et al*., 2001). Entropy was also reported to give an indication of class discrimination. Once the most parsimonious latent class model was determined, associations between treatment barrier class membership and demographic/clinical characteristics were explored using bivariate analyses. To examine the variables that best predict latent class membership, a multivariable logistic regression model was then built by including those predictor variables of latent class membership that were statistically significant in bivariate analyses (at *P* < 0.05). Age and sex were included as covariates in the final model. As missingness was extremely low (i.e. ≤0.3% for all variables), an available-case approach to missingness was appropriate. A *P*-value of <0.05 (two-sided) was used as the level of significance for statistical analyses, performed using Stata Version 15, Mplus Version 8 and IBM SPSS Statistics Version 25.

## RESULTS

Of 411 individuals screened for the parent trial, 344 were randomized and included in this study. Fifty-eight people were excluded from participating based on the study’s eligibility criteria, and nine eligible individuals were unable to be contacted for randomization. Individuals not eligible for participation reported significantly higher AUDIT total scores (*M* = 24.09, SD = 9.59) than individuals randomized to the study (*M* = 21.54, SD = 6.35; *F*(1,385) = 5.414, *P* = 0.020), and were more likely to have previously sought alcohol treatment (47 [71.21%] compared to 101 [29.36%]; χ^2^ = 42.05, *P* < 0.001). There were no differences between groups regarding other demographic or clinical variables. Individuals not eligible for participation were referred to other appropriate support when required.

### Demographic and clinical characteristics

Demographic and clinical characteristics are presented in Table 1. Participants had a mean age of 39.86 years (SD = 11.36, range 18–73 years), and just over half were male; there were 28 (8.14%) culturally and linguistically diverse participants, and 9 participants (2.62%) identified as being of Aboriginal and/or Torres Strait Islander descent. While two-thirds of participants lived in a metropolitan area, the rate of participation by geographic area was higher for non-metropolitan (1.6 per 100,000 population) than for metropolitan areas (1.3 per 100,000 population). Participants consumed alcohol an average of 19.90 (SD = 8.05) days in the past month, with an average of 15.45 (SD = 9.10) heavy drinking days (i.e. >4 standard drinks consumed) in the past month; mean alcohol problem severity based on the AUDIT was 21.54 (SD = 6.30) and nearly two-thirds (218, 63.37%) had a score in the highest symptom category of probable dependence. Approximately one-third of participants (130, 37.79%) had a K10 score in the symptom category of no significant psychological distress (mild psychological distress*,* 104, 30.23%; moderate psychological distress, 72, 20.93%; severe psychological distress, 38, 11.05%). In the past month, nearly half of participants (155, 45.60%) had used tobacco, one in five participants (73, 21.22%) had used cannabis, and use of other substances (e.g. amphetamine-type stimulants, cocaine) were each reported by <10% of the sample.

**Table 1:** Demographic and clinical characteristics, and associations with treatment barrier class membership

Variable	Total sample (*N* = 344) Count (%)	Low problem recognition class (*n* = 149) % (CI)	Complex barriers class (*n* = 195) % (CI)	Cohen’s *d*/odds ratio (CI)
Age (years), *M* (SD)	39.86 (11.36)	40.4 (38.57, 42.24)	39.45 (37.85, 41.05)	−0.08 (−0.30, 0.13)
Female sex	167 (48.55)	38.93 (31.10., 46.76)	55.90 (48.93, 65.87)	1.99^*^^*^ (1.29, 3.07)
Living in a metropolitan area	230 (66.86)	65.78 (58.15, 73.39)	67.69 (61.13, 74.26)	1.09 (0.69, 1.71)
Education				
<Year 12	39 (11.34)	12.75 (7.40, 18.11)	10.26 (6.00, 14.51)	0.78 (0.40, 1.52)
Year 12 or equivalent	60 (17.44)	18.79 (12.52, 25.06)	16.41 (11.21, 21.61)	0.85 (0.49, 1.48)
Vocational training, apprenticeship, Certificate	66 (19.18)	18.79 (12.52, 25.06)	19.49 (13.93, 25.05)	1.05 (0.61, 1.80)
Diploma, Advanced diploma, Associate degree	65 (18.90)	20.13 (13.70, 26.57)	17.95 (12.56, 23.34)	0.87 (0.50, 1.49)
Undergraduate degree	71 (20.64)	15.44 (9.64, 21.24)	24.62 (18.57, 30.66)	1.79^*^ (1.03, 3.10)
Postgraduate degree	43 (12.50)	14.09 (8.51, 19.68)	11.28 (6.84, 15.72)	0.78 (0.41, 1.47)
Employment status				
Full-time	151 (43.90)	44.97 (36.98, 52.95)	43.08 (36.13, 50.03)	0.95 (0.50, 1.78)
Part-time	56 (16.27)	14.77 (9.07, 20.46)	17.44 (12.11, 22.76)	0.93 (0.60, 1.42)
Casual	65 (18.89)	16.78 (10.78, 22.78)	20.51 (14.85, 26.18)	1.22 (0.68, 2.19)
Studying	14 (4.07)	4.70 (1.30, 8.10)	3.59 (0.98, 6.20)	1.28 (0.74, 2.22)
Retired	13 (3.78)	5.37 (1.75, 8.99)	2.56 (0.35, 4.78)	0.76 (0.26, 2.20)
Not employed	45 (13.08)	13.42 (7.95, 18.90)	12.82 (8.13, 17.51)	0.46 (0.15, 1.45)
Age first consumed alcohol, *M* (SD)	15.16 (2.52)	14.93 (14.52, 15.33)	15.34 (14.98, 15.69)	0.16 (−0.05, 0.38)
Age commenced regular alcohol consumption, *M* (SD)	18.23 (5.39)	18.21 (17.34, 19.08)	18.25 (17.48, 19.01)	0.01 (−0.21, 0.22)
Previous alcohol treatment	101 (29.36)	23.49 (16.68, 30.30)	33.85 (27.2, 40.49)	1.67^*^ (1.03, 2.70)
Alcohol problem severity (AUDIT), *M* (SD)	21.54 (6.30)	20.14 (19.13, 21.15)	22.61 (21.73, 23.49)	0.39^*^^*^^*^ (0.18, 0.60)
Past-month (30-days) alcohol consumption (TLFB), *M* (SD)				
Number of drinking days	19.90 (8.05)	19.98 (18.68, 21.28)	19.84 (18.7, 20.97)	−0.02 (−0.23, 0.20)
Days consuming >2 standard drinks	18.48 (8.36)	18.34 (16.99, 19.69)	18.58 (17.40, 19.77)	0.03 (−0.19, 0.24)
Days consuming >4 standard drinks	15.45 (9.10)	15.2 (13.73, 16.67)	15.64 (14.36, 16.92)	0.05 (−0.17, 0.26)
Total number of standard drinks	168.72 (108.16)	165.59 (148.14, 183.04)	171.12 (155.87, 186.37)	0.05 (−0.16, 0.27)
Psychological distress (K10), *M* (SD)	21.73 (6.02)	19.36 (18.45, 20.28)	23.51 (22.71, 24.31)	0.69^*^^*^^*^ (0.49, 0.89)

Chi-squared test for overall effect of education χ^2^(5) = 4.89, *P* = 0.430; chi-squared test for overall effect of employment status χ^2^(5) = 3.02, *P* = 0.697. Cohen’s *d* reported for class differences on continuous variables. Odd ratios represent odds of endorsing item in complex barriers class relative to low problem recognition class. Abbreviations: TLFB, Timeline Follow-back.

^^*^^ *P* < 0.05.

^^*^^*^^ *P* < 0.01.

^^*^^*^^*^^ *P* < 0.001.

### Previous alcohol treatment

Fewer than one-third of participants (101, 29.36%) had previously sought alcohol treatment. Among those who reported previous alcohol treatment, counselling (43, 12.50%), support and case management (e.g. GP advice, support and/or referral; 27, 7.85%) and pharmacotherapy (e.g. naltrexone, acamprosate, diazepam; 19, 5.52%,) were most frequently reported. The average number of treatment episodes reported was 1.33 (SD = 0.55, range 1–3).

#### Barriers to seeking treatment for alcohol problems

Participants endorsed multiple barriers to seeking treatment for alcohol problems (mean 5.64 barriers, SD = 2.41) and most frequently endorsed barriers belonging to attitudinal and readiness for change domains (Table 2). The three most frequently endorsed treatment barriers were (a) ‘*Thought I should be strong enough to handle it alone’* (282, 81.98%), (b) *‘Didn’t think drinking problem was serious enough’* (273, 79.36%), and (c) *‘Wanted to keep drinking’* (247, 71.80%).

**Table 2:** Frequency of perceived barriers to seeking treatment for alcohol problems

Treatment barrier (barrier domain)	Count (%)
Thought I should be strong enough to handle it alone (attitudinal)	282 (81.97)
Didn't think drinking problem was serious enough (readiness for change)	273 (79.36)
Wanted to keep drinking (readiness for change)	247 (71.80)
Thought the problem would get better by itself (attitudinal)	226 (65.70)
Was too embarrassed to discuss it with anyone (stigma)	166 (48.26)
Stopped drinking on my own (readiness for change)	147 (42.73)
Afraid of what friends, family, or others would think (stigma)	141 (40.99)
Didn't know where to go for help (structural)	104 (30.23)
Didn't have time (structural)	93 (27.03)
Didn't think anyone could help (attitudinal)	92 (26.74)
Couldn't afford to pay the bill (financial)	54 (15.70)
Was afraid I would lose my job (stigma)	38 (11.05)
Was afraid they would put me in hospital (attitudinal)	31 (9.01)
Wanted to go, but not covered by health insurance (financial)	24 (6.98)
Didn't have any way to get there (structural)	15 (4.36)

### Latent class analysis model

Indices of model fit were compared across one-, two-, three-, four- and five-class models (Table 3). The AIC and aBIC were not found to have a low point and thus were not useful in indicating model fit. Whilst the LMR p-value provided some evidence that a three-class model was better fitting than a two-class model (*P* = 0.012), there was a clear inflection point using BIC with the two-class model found to have the best fit. Given these results, the two-class model was determined to be the most optimal and parsimonious fit for the data. The retained two-class model had an entropy of 0.749, suggesting reliable class differentiation.

**Table 3:** Model fit indices for barriers to alcohol treatment of one- to five-class solutions

No. of classes	Log Likelihood	AIC	BIC	aBIC	LMR *p*-value	Entropy
T1 full sample						
1	-2600.77	5231.53	5289.14	5241.56	–	–
2	-2461.75	4985.5	5104.56	5006.22	<0.001	0.749
3	-2425.12	4944.23	5124.74	4975.65	0.012	0.81
4	-2388.81	4903.62	5145.58	4945.72	0.056	0.839
5	-2365.76	4889.52	5192.93	4942.33	0.059	0.851

### Latent class profiles

**Fig. 1:**
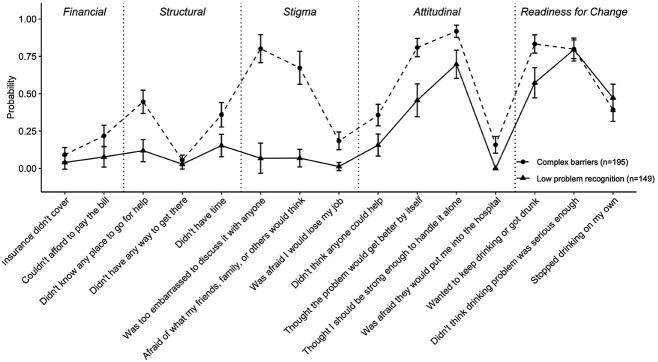
Weighted probability of endorsing treatment barriers by individuals presenting to telephone-delivered alcohol treatment, by latent class (two-class model). Note: a statistically significant difference between classes in terms of their probability of endorsing a barrier denoted by 95% confidence intervals that do not overlap.

Class one comprised 149 (43.3%) participants, and was operationalized as the ‘low problem recognition’ class. Class two comprised 195 (56.7%) participants, and was operationalized as the ‘complex barriers’ class (Fig. 1).

Participants in the complex barriers class endorsed a significantly greater number of barriers (*M* = 7.13, SD = 1.76) than participants in the low problem recognition class (*M* = 3.68, SD = 1.63; *F*(1,342) = 344.02, *P* < 0.001). Nearly all barrier items were relatively more likely to be endorsed by the complex barriers class than the low problem recognition class (Fig. 1).

Participants belonging to the low problem recognition class had a moderate-to-high probability of endorsing most barriers in two domains: readiness for change and attitudinal barriers. Barriers in these two domains are commonly reported by alcohol use populations and taken together are considered to indicate low problem awareness and perceived treatment need (May and Nielsen, 2019). The low problem recognition class had a very low probability of endorsing stigma, structural and financial barriers to alcohol treatment (Fig. 1). Participants belonging to the complex barriers class had a moderate-to-high probability of endorsing most barriers in four domains: attitudinal, readiness for change, stigma, and structural barriers. The complex barriers class had a low probability of endorsing financial barriers to alcohol treatment (Fig. 1).

### Characteristics associated with treatment barrier class membership

Bivariate analyses exploring associations between treatment barrier class membership and demographic and clinical characteristics (Table 1) found statistically significant associations between class membership and sex, previous alcohol treatment, alcohol problem severity, and psychological distress. In multivariable logistic regression (Table 4) adjusting for the predictor variables that were statistically significant in bivariate analyses (age was also included as a covariate in the final model), complex barriers class membership was predicted by female sex and higher levels of psychological distress.

**Table 4:** Multivariable logistic regression analysis for predictors of complex barrier class membership

Predictor	OR	95% CI
Age	1.00	0.98, 1.02
Sex (male = 0, female = 1)	2.23^*^^*^	1.38, 3.58
Previous AOD treatment (no = 0, yes = 1)	1.05	0.60, 1.84
Alcohol problem severity (AUDIT)	1.03	0.99, 1.08
Psychological distress (K10)	1.13^*^^*^^*^	1.08, 1.18

^^*^^*^^ *P* < 0.01.

^^*^^*^^*^^ *P* < 0.001.

## DISCUSSION

Of individuals with alcohol use problems from the general population presenting to a trial of telephone-delivered treatment, the majority (70.64%) were first-time help-seekers, despite two-thirds experiencing high levels of alcohol problem severity. This finding exceeds estimates from previous research that approximately one-third of clients accessing telephone-delivered alcohol interventions experience high alcohol problem severity (Heinemans *et al*., 2014). While telehealth services are considered an initial point of contact with the alcohol treatment system, and aim to facilitate earlier intervention, our results show that offering support by telephone attracted a larger proportion of people with high alcohol problem severity who had not previously accessed treatment, suggesting that this format of treatment may be overcoming barriers to care for this at-risk group.

In the current study, nearly half of participants accessing telephone-delivered alcohol treatment were female. This is in contrast to Australian alcohol treatment data that show two-thirds of clients accessing alcohol treatment are male (Australian Institute of Health and Welfare [AIHW], 2021), and suggests that telehealth may be filling a gap in service provision for women, who experience heightened barriers due to social stigma, complex trauma, childcare responsibilities and/or child custody concerns (Walter *et al*., 2003; Garde *et al*., 2017; Grigg *et al*., 2022). This finding, along with evidence of gender-specific aspects of problem alcohol consumption (Brienza and Stein, 2002) and increasing rates of alcohol consumption among women in some age groups (McCaul *et al*., 2019; AIHW, 2020), make a case for tailoring telephone-delivered alcohol treatment programs to meet the specific needs of women. Additionally, a higher rate of participation was observed among individuals located in non-metropolitan areas, compared to metropolitan areas. This finding contributes to the growing body of research showing the relatively greater uptake of telephone-delivered alcohol and other drug services among people living in regional and remote areas (Grigg *et al*., 2020, 2022), who are disproportionately affected by alcohol use and related harms (AIHW, 2020), yet receive fewer episodes of treatment (AIHW, 2019) and may experience heightened barriers to accessing treatment (e.g. lack of anonymity in smaller communities, geographical distance to services) (May and Nielsen, 2019).

Latent class analysis identified two distinct profiles of participants in terms of the barriers to seeking treatment: (a) a low problem recognition class, which was more likely to endorse readiness for change and attitudinal barriers, and (b) a complex barriers class, which was differentiated by a higher number of barriers endorsed (approximately twice that of the low problem recognition class) and endorsed attitudinal, readiness for change, and also stigma and structural barriers. These findings are partially consistent with previous research that has found classes differentiated by high and low frequency of barriers experienced by non-treatment seeking individuals with alcohol problems (Schuler *et al*., 2015). However, this is the first known study to examine barriers to help-seeking experienced by people presenting for telephone-delivered alcohol treatment. Results indicate two distinct barrier profiles for which the telephone format of treatment could be helping to overcome, including a low problem recognition subgroup, and a large subgroup experiencing a more complex set of barriers across multiple domains. Stigma about problem alcohol use persists as a major barrier to alcohol problem recognition and help-seeking (May and Nielsen, 2019). In the present study, stigma-related barriers were endorsed predominantly by the complex barriers class. Similarly, structural barriers were endorsed only by the complex barriers class. These results provide a more nuanced understanding of barriers to alcohol treatment than prevalence data permit by demonstrating subgroups that perceive and experience different treatment barriers, for whom differential intervention strategies (e.g. increasing problem recognition, therapies to address shame associated with problem alcohol use) should be explored.

Complex barrier class membership was predicted by female sex and higher psychological distress. This is consistent with previous research that has found people with alcohol and psychological comorbidity experience heightened barriers to treatment (Kaufmann *et al*., 2014). Additionally, while men are more likely than women to experience alcohol use problems (AIHW, 2020), women are more likely to face multiple barriers to accessing treatment (Walter *et al*., 2003; Garde *et al*., 2017) and may be at risk of adverse physiological and psychological health outcomes at lower drinking levels (Walter *et al*., 2003). Taken together, these findings provide important information on a large subgroup of participants for whom telephone-delivered alcohol intervention may be filling a critical unmet need. Findings point to the need for telephone-delivered models of alcohol treatment that integrate gender-specific and mental health treatment approaches.

The belief that one should be able to handle the problem alone is consistently identified as a prominent barrier preventing access to alcohol treatment among non-treatment seekers (Oleski *et al*., 2010; Schuler *et al*., 2015; May and Nielsen, 2019), as well as access to treatment for mental health problems more broadly (Prins *et al*., 2011); this was the barrier most frequently reported by participants in this study, and was more likely to be endorsed by the complex barriers class. Also consistent with previous research (Probst *et al*., 2015; Schuler *et al*., 2015), the belief that the problem is not severe enough to warrant treatment was a prominent barrier among the current sample, despite the high level of alcohol problem severity observed. Some research has suggested that people with alcohol use problems are motivated to maintain low problem recognition to avoid threat to the self-concept from identifying as a problem drinker, reinforced by the social stigma of alcoholism (Morris *et al*., 2021). Low problem awareness may also be driven by the prevailing social norms of heavy alcohol use specific to Western culture, as well as prolific (and exceedingly innovative) alcohol marketing and the frequent glamourization of alcohol consumption in media, which promote a culture of alcohol use where positive outcomes are anticipated (e.g. celebration, social connection, attractiveness) and the potential for harms are minimized or ignored. Public health strategies that counter this normalization of drinking to shift perceptions and raise awareness that even low levels of drinking cause harm/constitute problem alcohol use – and promoting alternative, easily accessible and less intensive treatment options such as telephone-delivered interventions—may increase help-seeking (Oleski *et al*., 2010; Probst *et al*., 2015), particularly among the population who are already at high risk of alcohol harms but are yet to perceive a need for treatment (Probst *et al*., 2015).

### Strengths and limitations

The data utilized in this study was drawn from a treatment-seeking sample recruited to an alcohol treatment trial; data collection was rigorous and standardized, and the dataset was reviewed and cleaned prior to analysis. LCA is a rigorous model-based approach that provides several fit statistics to assess model fit, and is appropriate for studying and classifying heterogeneity within a population. However, the cross-sectional nature of the data precludes the temporal ordering of variables, and it is recognized that class membership could change over alcohol consumption and help-seeking trajectories. Data on participants’ perceived treatment barriers were collected using binary indicator variables belonging to five pre-defined domains used in previous research (Grant *et al*., 2004; Schuler *et al*., 2015). While barrier domains were upheld in the current study, domain constructs could be refined in future studies (e.g. readiness for change domain does not align consistently with the Transtheoretical Model of Behaviour Change) (Prochaska and DiClemente, 1982). The question eliciting information on barriers to help-seeking did not specifically refer to barriers experienced in the context of no treatment, and as such this question may also have elicited information on barriers experienced in the context of receiving treatment (i.e. treatment was accessed despite barriers being experienced); barriers experienced in the context of treatment may be conceptually different and should be considered in future research. Further, barrier items were originally developed to identify barriers to alcohol treatment in the US population and some items may not have been as relevant to our Australian cohort (e.g. financial barriers: the majority of alcohol treatment services in Australia are publicly funded and available at no cost to the individual). However, this item may still have potential relevance as campaigns to promote these publicly-funded services are lacking and many individuals wait months to commence treatment (Health Complaints Commissioner, 2021). Barriers to alcohol treatment were reported by a treatment-seeking group with the findings not generalizable to the sizeable hidden population not presenting for treatment. Barriers to help-seeking vary considerably according to personal, cultural, and socio-economic factors; this was an Australian sample and the majority of participants were employed, with tertiary education beyond Year 12, and meeting inclusion for the parent trial, which limits the generalizability of the results including the latent class structure observed. While the telephone format of treatment delivery has the potential to overcome many of the treatment barriers reported by participants, no direct relationship between perceived barriers and the uptake of telephone treatment can be inferred from this study; it is recognized that other factors can also be influential in seeking help (e.g. disruptions to health or relationships, fluctuating motivation) (May and Nielsen, 2019). Additionally, there are likely specific barriers to telephone-delivered alcohol treatment (e.g. difficulty in achieving privacy to participate in a telephone session) that were not assessed by the current study. Indeed, barriers and facilitators of treatment likely vary across alcohol consumption and treatment-seeking trajectories, presenting multiple areas of future investigation to increase identification of treatment need, help-seeking, and treatment uptake.

## CONCLUSION

The majority of people presenting to telephone-delivered treatment for alcohol use problems were new to treatment, yet had high alcohol problem severity. More needs to be done to engage people with these easily accessible interventions earlier in their drinking trajectories. Participants had experienced multiple barriers to alcohol treatment, with the most strongly endorsed barrier being the attitudinal barrier, *‘Thought I should be strong enough to handle it alone’*. LCA identified two profiles regarding barriers to help-seeking, a ‘low problem recognition’ class, and a ‘complex barriers’ class (predicted by female sex and higher psychological distress), for which telephone interventions may overcome barriers to care and tailored approaches should be explored (e.g. increasing problem awareness, reducing psychological distress). Public health strategies to address stigma, and raise awareness about the low levels of drinking that constitute problem alcohol use, are needed to increase help-seeking.

## Data Availability

Data are not shared as there is no participant permission or Ethics approval to do so, other than for related projects conducted by the research team.

## References

[ref1] Akaike H . (1987) Factor analysis and AIC. In Parzen E, Tanabe K, Kitagawa G (eds). Selected Papers of Hirotugu Akaike. New York, NY: Springer New York, 371–86.

[ref2] Australian Bureau of Statistics (ABS) . (2018) The Australian Statistical Geography Standard (ASGS) Remoteness Structure. Canberra, Australia: ABS.

[ref3] Australian Institute of Health and Welfare . (2020) National Drug Strategy Household Survey 2019. Canberra, Australia: AIHW.

[ref4] Australian Institute of Health and Welfare . (2021) Alcohol and Other Drug Treatment Services in Australia: Key Findings. Canberra, Australia: AIHW.

[ref5] Australian Institute of Health and Welfare (AIHW) . (2019) Alcohol and Other Drug Treatment Services in Australia 2017–18: Key Findings. Canberra, Australia: AIHW.

[ref6] Australian Institute of Health and Welfare (AIHW) . (2020) Daniel F and Ian PA (eds). Alcohol, Tobacco & Other Drugs in Australia. Cat. No: PHE 221. Canberra, Australia: AIHW.

[ref7] Babor TF , Higgins-Biddle J, Saunders JB et al. (2001) The Alcohol Use Disorders Identification Test: Guidelines for Use in Primary Care. Geneva, Switzerland: World Health Organization.

[ref8] Bernard C , Grigg J, Volpe I et al. (2020) Client experiences of a telephone-delivered intervention for alcohol use: a qualitative study. Int J Ment Health Addiction 20:522–40.

[ref9] Brienza RS , Stein MD. (2002) Alcohol use disorders in primary care: do gender-specific differences exist? J Gen Intern Med 17:387–97. 12047738 10.1046/j.1525-1497.2002.10617.x PMC1495039

[ref10] Chapman C , Slade T, Hunt C et al. (2015) Delay to first treatment contact for alcohol use disorder. Drug Alcohol Depend 147:116–21. 25533894 10.1016/j.drugalcdep.2014.11.029

[ref11] Garde EL , Manning V, Lubman DI. (2017) Characteristics of clients currently accessing a national online alcohol and drug counselling service. Australas Psychiatry 25:250–3. 28541729 10.1177/1039856216689623

[ref12] Gates P , Albertella L. (2016) The effectiveness of telephone counselling in the treatment of illicit drug and alcohol use concerns. J Telemed Telecare 22:67–85. 26026185 10.1177/1357633X15587406

[ref13] Grant BF , Stinson FS, Dawson DA et al. (2004) Prevalence and co-occurrence of substance use disorders and independentmood and anxiety disorders: results from the national epidemiologic survey on alcohol and relatedconditions. Arc Gen Psychiatry 61:807–16. 10.1001/archpsyc.61.8.807 15289279

[ref14] Grigg J , Arunogiri S, Manning V et al. (2020) The Drug and Alcohol Clinical Advisory Service: a model of telephone-delivered addiction specialist support. Drug Alcohol Rev 39:238–45. 31972876 10.1111/dar.13035

[ref15] Grigg J , Volpe I, Tyler J et al. (2022) Ready2Change: preliminary effectiveness of a telephone-delivered intervention program for alcohol, methamphetamine and cannabis use problems. Drug Alcohol Rev 41:517–52. 34343370 10.1111/dar.13363

[ref16] Griswold MG , Fullman N, Hawley C et al. (2018) Alcohol use and burden for 195 countries and territories, 1990–2016: a systematic analysis for the Global Burden of Disease Study 2016. Lancet 392:1015–35. 30146330 10.1016/S0140-6736(18)31310-2 PMC6148333

[ref17] Harris PA , Taylor R, Minor BL et al. (2019) The REDCap consortium: building an international community of software platform partners. J Biomed Inform 95:103208. 31078660 10.1016/j.jbi.2019.103208 PMC7254481

[ref18] Health Complaints Commissioner . (2021) Review of Private Health Service Providers Offering AOD Rehabilitation and Counselling Services in Victoria Melbourne. Australia: Victoria State Government.

[ref19] Heinemans N , Toftgård M, Damström-Thakker K et al. (2014) An evaluation of long-term changes in alcohol use and alcohol problems among clients of the Swedish National Alcohol Helpline. Subst Abuse Treat Prev Policy 9:1–9. 24893718 10.1186/1747-597X-9-22 PMC4055694

[ref20] Kaufmann CN , Chen L-Y, Crum RM et al. (2014) Treatment seeking and barriers to treatment for alcohol use in persons with alcohol use disorders and comorbid mood or anxiety disorders. Soc Psychiatry Psychiatr Epidemiol 49:1489–99. 23900549 10.1007/s00127-013-0740-9 PMC3983167

[ref21] Kessler RC , Berglund P, Demler O et al. (2003) The epidemiology of major depressive disorder: results from the National Comorbidity Survey Replication (NCS-R). JAMA 289:3095–105. 12813115 10.1001/jama.289.23.3095

[ref22] Lin LA , Casteel D, Shigekawa E et al. (2019) Telemedicine-delivered treatment interventions for substance use disorders: a systematic review. J Subst Abuse Treat 101:38–49. 31006553 10.1016/j.jsat.2019.03.007

[ref23] Lo Y , Mendell NR, Rubin DB. (2001) Testing the number of components in a normal mixture. Biometrika 88:767–78.

[ref24] Lubman DI , Grigg J, Manning V et al. (2019) A structured telephone-delivered intervention to reduce problem alcohol use (Ready2Change): study protocol for a parallel group randomised controlled trial. Trials 20:1–12. 31426835 10.1186/s13063-019-3462-9 PMC6701125

[ref25] May C , Nielsen AS. (2019) Barriers to treatment for alcohol dependence. J Drug Alcohol Res 8:1–17.

[ref26] McCaul ME , Roach D, Hasin DS et al. (2019) Alcohol and women: a brief overview. Alcohol Clin Exp Res 43:774. 30779446 10.1111/acer.13985 PMC6502688

[ref27] Morris J , Albery IP, Moss AC et al. (2021) Promoting problem recognition amongst harmful drinkers: a conceptual model for problem framing factors. In The Handbook of Alcohol Use. London, United Kingdom: Elsevier, 221–36.

[ref28] Murray CJ , Aravkin AY, Zheng P et al. (2020) Global burden of 87 risk factors in 204 countries and territories, 1990–2019: a systematic analysis for the Global Burden of Disease Study 2019. Lancet 396:1223–49. 33069327 10.1016/S0140-6736(20)30752-2 PMC7566194

[ref29] Ogeil RP , Scott D, Faulkner A et al. (2021) Changes in alcohol intoxication-related ambulance attendances during COVID-19: how have government announcements and policies affected ambulance call outs? Lancet Reg Health West Pac 14:100222. 34545354 10.1016/j.lanwpc.2021.100222 PMC8443417

[ref30] Oleski J , Mota N, Cox BJ et al. (2010) Perceived need for care, help seeking, and perceived barriers to care for alcohol use disorders in a national sample. Psychiatr Serv 61:1223–31. 21123407 10.1176/ps.2010.61.12.1223

[ref31] Pollard MS , Tucker JS, Green HD. (2020) Changes in adult alcohol use and consequences during the COVID-19 pandemic in the US. JAMA Netw Open 3:e2022942–e42. 32990735 10.1001/jamanetworkopen.2020.22942 PMC7525354

[ref32] Prins M , Meadows G, Bobevski I et al. (2011) Perceived need for mental health care and barriers to care in the Netherlands and Australia. Soc Psychiatry Psychiatr Epidemiol 46:1033–44. 20686887 10.1007/s00127-010-0266-3 PMC3173635

[ref33] Probst C , Manthey J, Martinez A et al. (2015) Alcohol use disorder severity and reported reasons not to seek treatment: a cross-sectional study in European primary care practices. Subst Abuse Treat Prev Policy 10:1–10. 26264215 10.1186/s13011-015-0028-z PMC4534056

[ref34] Prochaska JO , DiClemente CC. (1982) Transtheoretical therapy: toward a more integrative model of change. Psychother Theor Res 19:276.

[ref35] Rehm J , Shield KD, Rehm MX et al. (2012) Alcohol Consumption, Alcohol Dependence, and Attributable Burden of Disease in Europe: Potential Gains from Effective Interventions for Alcohol Dependence, Report 1771140461. Canada: Centre for Addiction and Mental Health.

[ref36] Sallie SN , Ritou V, Bowden-Jones H et al. (2020) Assessing international alcohol consumption patterns during isolation from the COVID-19 pandemic using an online survey: highlighting negative emotionality mechanisms. BMJ Open 10:e044276. 10.1136/bmjopen-2020-044276 PMC7692002 33243820

[ref37] Schuler MS , Puttaiah S, Mojtabai R et al. (2015) Perceived barriers to treatment for alcohol problems: a latent class analysis. Psychiatr Serv 66:1221–8. 26234326 10.1176/appi.ps.201400160 PMC4630073

[ref38] Schwarz G . (1978) Estimating the dimension of a model. Ann Stat 6:461–4.

[ref39] Sobell LC , Sobell MB. (1992) Timeline follow-back. In Litten RZ, Allen JP (eds). Measuring Alcohol Consumption. Totowa, NJ: Humana Press, 41–72.

[ref40] Stead LF , Buitrago D, Preciado N et al. (2013) Physician advice for smoking cessation. Cochrane Database Syst Rev 5:CD000165. 10.1002/14651858.CD000165.pub4 PMC7064045 23728631

[ref41] Sterne JA , Hernán MA, Reeves BC et al. (2016) ROBINS-I: a tool for assessing risk of bias in non-randomised studies of interventions. BMJ 355:i4919. 10.1136/bmj.i4919 PMC5062054 27733354

[ref42] Von Elm E , Altman DG, Egger M et al. (2007) The Strengthening the Reporting of Observational Studies in Epidemiology (STROBE) statement: guidelines for reporting observational studies. Ann Intern Med 147:573–7. 17938396 10.7326/0003-4819-147-8-200710160-00010

[ref43] Walter H , Gutierrez K, Ramskogler K et al. (2003) Gender-specific differences in alcoholism: implications for treatment. Arch Womens Ment Health 6:253–8. 14628177 10.1007/s00737-003-0014-8

[ref44] World Health Organization . (2018) Global Status Report on Alcohol and Health 2018. Geneva, Switzerland: World Health Organization.

[ref45] World Health Organization . (2022) Alcohol. Geneva, Switzerland: World Health Organization.

